# A Completed Cycle Audit of a Tongue-Tie Management Pathway for Newborns

**DOI:** 10.7759/cureus.95822

**Published:** 2025-10-31

**Authors:** Alicia Wong, Manuela Cresswell, Mihiar Atfeh

**Affiliations:** 1 Ear, Nose, and Throat, University Hospitals Plymouth NHS Trust, Plymouth, GBR; 2 Otolaryngology, University Hospitals Plymouth NHS Trust, Plymouth, GBR

**Keywords:** ankyloglossia, audit, breastfeeding, frenulotomy, primary care, referral pathway, tongue-tie

## Abstract

Introduction: Tongue-tie (ankyloglossia) can cause significant feeding difficulties in infants. A quality improvement project conducted in 2015-2016 at our centre led to the introduction of a referral pathway that standardised frenulotomy (tongue-tie release) services. This project completes a second-cycle audit to evaluate the appropriateness and effectiveness of the tongue-tie referral pathway for patients under three months old.

Methods: A retrospective case-note review was conducted on 310 patients referred for tongue-tie assessment between January 2019 and December 2023. Data were collected from hard copies and electronic patient records. Parameters assessed included referral source, use of the proforma, use of the Bristol Tongue Assessment Tool (BTAT), and whether division was performed.

Discussion: In the final project cycle, 73.2% of tongue-tie referrals included a completed proforma. The number of patients undergoing frenulotomy increased from 53% prior to the pathway to 61% in the first cycle and then to 95.9% in the final cycle. There is an increase in referral appropriateness and in the correlation between referral indications and ENT clinic findings. A total of 10% of referrals lacked an attached clinical letter, and alternative or incomplete referral methods were still occasionally used. General practitioners (GPs) and advanced nurse practitioners (ANPs) were more likely to refer via the alternative systems rather than through the pathway.

Conclusion: The completed project confirms the effectiveness and appropriateness of the tongue-tie referral pathway, leading to more accurate referrals and higher intervention rates. Scope for improvement could include future updates of the proforma and providing further education to community referrers. Future reviews and integration with electronic systems and implementation of One Devon may enhance consistency and long-term sustainability.

## Introduction

Ankyloglossia, commonly known as a tongue-tie, is a congenital anomaly characterised by a short or thickened lingual frenulum that causes an adhesion of varying severity of the tongue tip to the floor of the mouth and can result in restricted tongue mobility [1]. This creates a heart-shaped appearance of the tongue and may affect feeding. It occurs in 4-10% of newborns [2], and it may lead to feeding difficulties such as poor latch while breastfeeding, clicking sounds, excessive intake of air, increased frequency of feeds, and failure to thrive. For the mother, it can cause breast trauma, reduced milk supply, and mastitis [3]. Although not all cases require intervention, parental concern regarding feeding and speech development often prompts referral.

At our centre, a quality improvement project in 2015-2016 identified that many tongue-tie referrals were inappropriate and did not require surgical division. This created unnecessary demand on the service and stress for families. In response, a structured referral pathway was introduced, incorporating a proforma with the Bristol Tongue Assessment Tool (BTAT), a validated clinical tool designed to provide an objective and simple assessment of tongue-tie in infants [4], with the aim of standardising community referrals, reducing inappropriate referrals, and improving efficiency. This pathway targets clinicians in primary care and maternity services with the aim of improving the tongue-tie referral pathway, reducing the volume of inappropriate referrals, and reducing the time from referral to specialist assessment to enhance efficiency and patient experience of the service. This study reports the final cycle of a completed audit assessing the appropriateness and effectiveness of the tongue-tie referral pathway for infants under three months.

## Materials and methods

Study design

This study represents the final phase of a multi-cycle quality improvement audit conducted at a single centre, University Hospitals Plymouth NHS Trust, United Kingdom. The audit evaluated the effectiveness of a structured referral pathway for infants with suspected ankyloglossia. The baseline audit cycle was completed in 2015, followed by a first post-intervention cycle, with the final audit cycle spanning January 2019 to December 2023. The project was registered with the local clinical audit department as part of ongoing service evaluation; therefore, formal research ethics approval was not required. 

Patient recruitment

All infants aged less than three months who were referred to the ENT service for tongue-tie assessment between January 2019 and December 2023 were included. Referrals originated from a range of community healthcare professionals, including midwives, health visitors, general practitioners (GPs), advanced nurse practitioners (ANPs), and paediatric doctors. 

Exclusion criteria 

The exclusion criteria were infants over three months of age at referral, with incomplete or missing clinical records preventing data extraction. A total of 310 eligible patient records were included in the final analysis. 

Data collection 

Data were extracted retrospectively from both paper and electronic paper records. Variables collected included demographic details (age at referral, sex), the referring clinician’s designation, completion and content of the referral proforma, documentation of the BTAT score, indication for referral, clinic findings, and whether a frenulotomy was performed.

Bristol Tongue Assessment Tool (BTAT)

The BTAT is a validated clinical instrument developed by Ingram et al. to provide an objective, reproducible assessment of tongue-tie severity in infants. It evaluates four domains: tongue tip appearance, attachment of the frenulum to the lower gum ridge, lift of the tongue with the mouth wide open (crying), and protrusion of the tongue beyond the lower gum. Each parameter is scored from 0 to 2, producing a total score ranging from 0 to 8, where lower scores indicate greater restriction of tongue movement [5].

The BTAT underwent validation through inter-rater reliability testing and comparison with the Hazelbaker Assessment Tool for Lingual Frenulum Function (HATLFF), demonstrating strong agreement (r = 0.89) and improved ease of use [5]. Unlike the HATLFF, which contains 14 criteria and is often considered too complex for routine practice, the BTAT provides a concise, objective, and reproducible assessment suitable for both community and specialist settings. Its brevity and simplicity enhance consistency and facilitate widespread adoption among multidisciplinary teams.

Statistical analysis

Data were collated and analysed using MS Excel (Microsoft Corporation, Redmond, Washington, United States). Descriptive statistics, including frequencies and percentages, were used to summarise demographic characteristics, referral sources, completion proformas, and BTAT scores [6]. Comparative analysis was performed against previous audit cycles (2015 and 2016-2018) to assess longitudinal improvement in pathway performance and the proportion of referrals resulting in frenulotomy. Additionally, a focused literature review was conducted using PubMed with the search terms ("ankyloglossia" or “tongue tie") AND ("infant" OR "neonate") AND "referral" to contextualise findings with existing evidence. 

Ethical considerations 

This project was conducted as part of a registered local clinical audit under the governance of the University Hospitals Plymouth NHS Trust. No direct patient contact occurred, and all data were anonymised prior to analysis. Therefore, institutional research ethics approval was not required. 

## Results

A total of 310 infants under three months of age were referred for tongue-tie assessment between January 2019 and December 2023, representing the final cycle of this quality improvement audit. Only infants under three months of age with complete referral and clinic documentation were included, while those with incomplete or missing records, or aged over three months at referral, were excluded. This ensured a consistent cohort and reduced the risk of selection bias in evaluating pathway effectiveness. The cohort comprised 165/310 (53.2%) males and 145/310 (46.8%) females. 

Frenulotomy rates 

Of the 310 referred infants, 297/310 (95.9%) underwent frenulotomy, representing a substantial increase compared with earlier audit cycles. At baseline, only 42/79 (53.2%) resulted in frenulotomy, followed by 26/49 (53.1%) in the first cycle and 19/31 (61.3%) in the second cycle. This progressive improvement indicates that post-pathway referrals were increasingly appropriate and clinically justified, demonstrating enhanced triage accuracy and diagnostic precision. 

Referral sources 

Health visitors were the most frequent referrers (103/310, 33.2%), followed by midwives (85/310, 27.4%), GPs (48/310, 15.5%), paediatric doctors (27/310, 8.7%), and ANPs (8/310, 2.6%). The referrer was unknown in 39/310 (12.6%) cases. Compared to the earlier cycles, a larger proportion of referrals in this final phase originated from health visitors and midwives, suggesting that community engagement and pathway awareness had improved since the implementation of the structured referral process. 

Indications for referral 

Feeding difficulties remained the predominant reason for referral (274/310, 88.4%), consistent with national patterns and the baseline audit findings. Only a small number of referrals cited dual concerns of feeding and speech (3/310, 1.0%), or other symptoms such as snoring in 1/310 (0.3%). In 31/310 (10%) cases, the referral form lacked a stated indication.

BTAT scores 

BTAT scores were available in 222/310 (71.6%) referrals. The most common scores were 4 in 67/310 (21.6%), 3 in 46/310 (14.8%), and 5 in 40/310 (12.9%), consistent with moderate tongue restriction. Severe restriction (score 0-2) was reported in 34/310 (11.0%), while 35/310 (11.3%) had functional scores greater than 6, suggesting minimal restriction. Compared to previous audit cycles, the current data show improved documentation and a higher proportion of infants with clinically significant restriction at referral, reinforcing the enhanced appropriateness of referrals following pathway implementation. Scores were not documented in 88/310 (28.4%) cases.

Trend summary 

Overall, the data demonstrated improvement across all key audit parameters. Frenulotomy was performed in 297/310 cases (95.9%) compared with 42/79 (53.2%) at baseline, indicating a substantial increase in referral appropriateness and clinical alignment. Use of the referral proforma rose to 227/310 (73.2%) from 37.8% in earlier cycles, reflecting improved pathway adherence. Documentation quality also improved, with the proportion of referrals lacking a stated indication decreasing by more than half compared to previous audits. The distribution of BTAT scores shifted towards moderate to severe restriction, with most scores between 3 and 5, suggesting enhanced pre-referral triage accuracy and more targeted clinical assessment. Collectively, these findings confirm that implementation of a structured referral pathway incorporating the BTAT has improved both efficiency and diagnostic precision in tongue-tie management for infants under three months of age.

## Discussion

This completed cycle audit demonstrates that the introduction of a structured referral pathway has significantly improved the clinical appropriateness and effectiveness of tongue-tie management in infants under three months of age. Key elements of the pathway included a standardised proforma incorporating the BTAT, enhanced staff training, and provision of patient information leaflets. Collectively, these interventions contributed to improved diagnostic precision and a higher proportion of referrals leading to frenulotomy. 

Following pathway implementation, the proportion of referrals resulting in frenulotomy increased from 53% at baseline to 95.9% in the final cycle, suggesting that referrals are now more closely aligned with clinical need and that primary care triage has become more discerning. Uptake of the proforma was high (73.2%), indicating good adoption across community referrers, although incomplete documentation remained a limitation. These findings are consistent with the existing literature. Baxter et al. described the development of a tongue restriction questionnaire focusing on elevation and symptom history, which classified most children as having only mild restriction [7]. In comparison, our cohort assessed the BTAT most commonly scored 3-5, also indicating moderate restriction. Both studies reinforce the variable clinical presentation of tongue-tie and highlight tongue elevation as a reliable proxy for function. Importantly, both emphasise the need for shared decision-making and careful assessment to avoid both under- and over-treatment, supporting the value of structured referral pathways and validated assessment tools.

Despite improvements, the literature underscores persistent challenges in tongue-tie diagnosis. The HATLFF, cited in the National Institute for Health and Care Excellence (NICE) guidelines [3], has been criticised as overly complex for routine use. A systematic review by Manipon [8] highlighted wide variation in diagnostic criteria and timing of assessment, contributing to reported prevalence rates ranging from 4.2% to 10.7%. These inconsistencies highlight the urgent need for a concise, validated, and universally accepted diagnostic tool, alongside clearer division frameworks for when frenulotomy is indicated. As breastfeeding remains the recommended method of infant nutrition, healthcare professionals have a responsibility to ensure equitable access to timely and evidence-based tongue-tie management [9]. 

Limitations

This audit has several limitations. The retrospective design relied on the accuracy and completeness of clinical documentation, and missing BTAT scores (28.4%) reduced the dataset available for functional analysis. This incomplete data capture may have introduced minor bias in estimating referral appropriateness and severity distribution. Future audit cycles could mitigate this limitation through prospective data collection using a standardised electronic form integrated within the patient record, ensuring automatic capture of BTAT scores and referral details. Additionally, triangulating information from multiple data sources, such as community midwifery and electronic referral systems, could improve completeness and data accuracy. As this was a single-centre study, findings may not be generalisable to other healthcare systems, though the structured approach may still be applicable across similar NHS settings.

Future directions

Embedding the referral pathway into an electronic patient record system, including One Devon, would promote consistency and facilitate real-time data collection [8]. Future audits should evaluate the sustainability of improvements and include outcomes such as breastfeeding rates and patient satisfaction. Ongoing training, feedback, and periodic updates to the proforma will help maintain engagement and ensure continued quality improvement. 

## Conclusions

Tongue-tie is a common congenital condition that may significantly affect infant feeding and parental well-being. Tongue-tie necessitates urgent outpatient appointments, which create pressure on service capacity and contribute to unnecessary stress and anxiety for the parents. Not all cases require intervention, but often tongue-tie prompts parental concerns regarding feeding and speech development during the neonatal period. This audit demonstrates that a structured referral pathway incorporating a standardised proforma and BTAT improved the appropriateness of referrals, diagnostic clarity, and intervention rates in infants under three months of age. The findings support the value of structured pathways in ensuring that infants requiring frenulotomy are more accurately identified, thereby reducing unnecessary referrals and improving service efficiency. Ongoing professional education, interprofessional collaboration, and shared decision-making remain central to optimising care. Development and adoption of a universally validated assessment tool would further enhance consistency and long-term sustainability.
